# Improving the low solubility of resveratrol

**DOI:** 10.1186/2050-6511-13-S1-A25

**Published:** 2012-09-17

**Authors:** Milica T Atanacković, Ljiljana C Gojković-Bukarica, Jelena M Cvejić

**Affiliations:** 1 Department of Pharmacy, Faculty of Medicine, 21000 Novi Sad, Serbia; 2 Institute of Pharmacology, Clinical Pharmacology and Toxicology, Medical Faculty, University of Belgrade, 11129 Belgrade, Serbia

## Background

Resveratrol, a polyphenol mainly present in grapes and red wine, demonstrated interesting biomedical properties for its cardioprotective action due to inhibition of the oxidation of low-density lipoprotein (LDL) and of platelet aggregation, inhibitory effects on cancer promotion and propagation and anti-inflammatory activities. These potential therapeutic and prophylactic applications are limited by the low bioavailability caused by its physical properties. Additionally, resveratrol has low water solubility and stability making its clinical success a formidable technological and medical challenge. The aim of this work is to present results of improvement of solubility of resveratrol through micellar and liposomal incorporation.

## Methods

Solubilization of resveratrol in six different bile acid solutions (cholic acid and its keto derivates) was investigated after 18 hours of mixing 2 mg of resveratrol in 2 ml of bile acid solutions in pH 7 buffer at room temperature. Liposome preparations containing pure resveratrol, resveratrol with vitamin C and resveratrol with vitamin E were prepared using the thin film hydration method. Resveratrol content was analyzed using HPLC with UV/DAD detection.

## Results

The analysis of solubilization of resveratrol showed that keto derivatives of cholic acid have greater ability to solubilize resveratrol than cholic acid, and that this efficiency increases with the number of keto groups present in bile acid. The most effective acid for the solubilization of resveratrol was 3,7,12-triketocholic acid. Also, it has been shown that the efficiency of incorporation of resveratrol in liposomes increases with the amount of added resveratrol during preparation and the presence of vitamin C or E in the formulation, and that these preparations have satisfactory characteristics.

## Conclusions

Experiments carried out in this study provide useful information for potential development of different dietary and pharmaceutical resveratrol products.

